# Non-Woven Infection Prevention Fabrics Coated with Biobased Cranberry Extracts Inactivate Enveloped Viruses Such as SARS-CoV-2 and Multidrug-Resistant Bacteria

**DOI:** 10.3390/ijms222312719

**Published:** 2021-11-24

**Authors:** Kazuo Takayama, Alberto Tuñón-Molina, Alba Cano-Vicent, Yukiko Muramoto, Takeshi Noda, José Luis Aparicio-Collado, Roser Sabater i Serra, Miguel Martí, Ángel Serrano-Aroca

**Affiliations:** 1 Center for iPS Cell Research and Application (CiRA), Kyoto University, Kyoto 606-8507, Japan; kazuo.takayama@cira.kyoto-u.ac.jp; 2 Biomaterials and Bioengineering Lab, Centro de Investigación Traslacional San Alberto Magno, Universidad Católica de Valencia San Vicente Mártir, c/Guillem de Castro 94, 46001 Valencia, Spain; alberto.tunon@ucv.es (A.T.-M.); alba.cano@mail.ucv.es (A.C.-V.); miguel.marti@ucv.es (M.M.); 3 Laboratory of Ultrastructural Virology, Institute for Frontier Life and Medical Sciences, Kyoto University, Kyoto 606-8507, Japan; muramo@infront.kyoto-u.ac.jp (Y.M.); t-noda@infront.kyoto-u.ac.jp (T.N.); 4 Centre for Biomaterials and Tissue Engineering, Universitat Politècnica de València, 46022 València, Spain; joapcol@upvnet.upv.es (J.L.A.-C.); rsabater@die.upv.es (R.S.i.S.); 5 CIBER-BBN, Biomedical Research Networking Centre in Bioengineering, Biomaterials and Nanomedicine, 46022 València, Spain

**Keywords:** SARS-CoV-2, enveloped viruses, bacteriophage phi 6, multidrug-resistant, bacteria, *Staphylococcus aureus*, *Staphylococcus epidermidis*, non-woven fabric, cranberry extract, antimicrobial activity, infection prevention clothing

## Abstract

The Coronavirus Disease (COVID-19) pandemic is demanding the rapid action of the authorities and scientific community in order to find new antimicrobial solutions that could inactivate the pathogen SARS-CoV-2 that causes this disease. Gram-positive bacteria contribute to severe pneumonia associated with COVID-19, and their resistance to antibiotics is exponentially increasing. In this regard, non-woven fabrics are currently used for the fabrication of infection prevention clothing such as face masks, caps, scrubs, shirts, trousers, disposable gowns, overalls, hoods, aprons and shoe covers as protective tools against viral and bacterial infections. However, these non-woven fabrics are made of materials that do not exhibit intrinsic antimicrobial activity. Thus, we have here developed non-woven fabrics with antimicrobial coatings of cranberry extracts capable of inactivating enveloped viruses such as SARS-CoV-2 and the bacteriophage phi 6 (about 99% of viral inactivation in 1 min of viral contact), and two multidrug-resistant bacteria: the methicillin-resistant *Staphylococcus aureus* and the methicillin-resistant *Staphylococcus epidermidis*. The morphology, thermal and mechanical properties of the produced filters were characterized by optical and electron microscopy, differential scanning calorimetry, thermogravimetry and dynamic mechanical thermal analysis. The non-toxicity of these advanced technologies was ensured using a *Caenorhabditis elegans* in vivo model. These results open up a new prevention path using natural and biodegradable compounds for the fabrication of infection prevention clothing in the current COVID-19 pandemic and microbial resistant era.

## 1. Introduction

Severe Acute Respiratory Syndrome Coronavirus 2 (SARS-CoV-2) is the third human coronavirus [1,2,3,4] that is much more contagious than SARS-CoV and MERS-CoV [5,6,7,8,9,10,11,12]. This rapid transmission rate has provoked the current Coronavirus Disease (COVID-19) pandemic. SARS-CoV-2 is a highly pathogenic enveloped positive-sense single-stranded RNA virus [13,14,15] that belongs to the Baltimore group IV [16]. This global life-threatening situation needs the development of new antimicrobial approaches that could treat or prevent COVID-19 infections [17,18,19,20,21,22]. In this regard, non-woven fabrics are currently used for the fabrication of infection prevention clothing such as face masks, caps, scrubs, shirts, trousers, disposable gowns, overalls, hoods, aprons, and shoe covers. These infection prevention tools are needed specially in hospitals during surgical operations, in microbiological and biomedical biosafety laboratories and also, in the case of face masks, by most citizens as a demonstrated prevention tool in the current COVID-19 pandemic [23]. Nevertheless, these infection prevention clothing are produced with materials that do not possess antimicrobial properties, and some progress has been achieved so far in the development of antimicrobial prevention fabrics [24]. Many disinfectants such as household bleach, hand soap solution, ethanol, povidone-iodine, chloroxylenol, chlorhexidine and benzalkonium chloride have shown potent antiviral activity against SARS-CoV-2 so far [25]. Thus, non-woven fabrics for the fabrication of face masks and face shields have been treated with benzalkonium chloride to produce antimicrobial infection prevention tools [26,27]. Very recently, non-woven fabrics have been coated with solidified hand soap to produce antimicrobial face masks capable of inactivating SARS-CoV-2 in one min of contact [28]. Other authors have produced next generation infection prevention materials using other antimicrobial agents such as antimicrobial polymers, salts, carbon nanomaterials, metals or metal oxides [24,29,30,31]. However, very few studies about the use of natural and biodegradable compounds such as cranberry extracts have been reported [24]. Cranberry extracts have shown antiviral activity against other enveloped viruses such as the herpes simplex virus type 1 (HSV-1) and type 2 (HSV-2) due to the presence of antimicrobial A-type proanthocyanidins (PACs) that provoke alterations of their envelope glycoproteins. However, although HSV-1 and HSV-2 belong to a different Baltimore group I [16] than SARS-CoV-2 because they are double-stranded DNA viruses, they are also enveloped viruses like SARS-CoV-2. A cranberry extract has also exhibited antiviral activity against influenza virus (IFV) [32]. IFV is a negative-sense single-stranded RNA virus that belongs to the Baltimore group V [16]. However, it is also enveloped like HSV-1 and HSV-2. Therefore, since it seems that the PACs present in cranberry extracts effectively interact with the envelope glycoproteins achieving viral inhibition, we hypothesize here that a commercial non-woven fabric treated with two different commercial extracts produced with different cranberries will show antiviral activity against the enveloped SARS-CoV-2 and bacteriophage phi 6. The bacteriophage phi 6 is also an enveloped double-stranded RNA virus (group III of the Baltimore classification [16]) that can be used as surrogate of SARS-CoV-2 and other enveloped viruses such as influenza due to biosafety reasons [26]. Furthermore, atypical viral pneumonia is associated with SARS-CoV-2 infection [2,33] and can increase its risk by co-infection with bacteria [34,35,36,37], including clinically relevant antibiotic-resistant strains. Additionally, bacterial resistance to pneumonia treatments is increasing at an alarming rate [38,39]. Since the PACs present in cranberry extracts are well-known for their antibacterial properties against Gram-negative *Escherichia coli* [40] and antifungal activity against *Candida albicans* [41], we hypothesize here also that the two non-woven fabrics dip-coated with cranberry extracts will show also antibacterial activity against two Gram-positive multidrug-resistant bacteria, the methicillin-resistant *Staphylococcus aureus* (MRSA) and the methicillin-resistant *Staphylococcus epidermidis* (MRSE). These advanced fabrics will be characterized by optical and electron microscopy, differential scanning calorimetry, thermogravimetry, dynamic mechanical thermal analysis and their toxicological aspects will be analyzed using a *Caenorhabditis elegans* in vivo model.

## 2. Results and Discussion

### 2.1. Fabric Morphology

**Figure 1:**
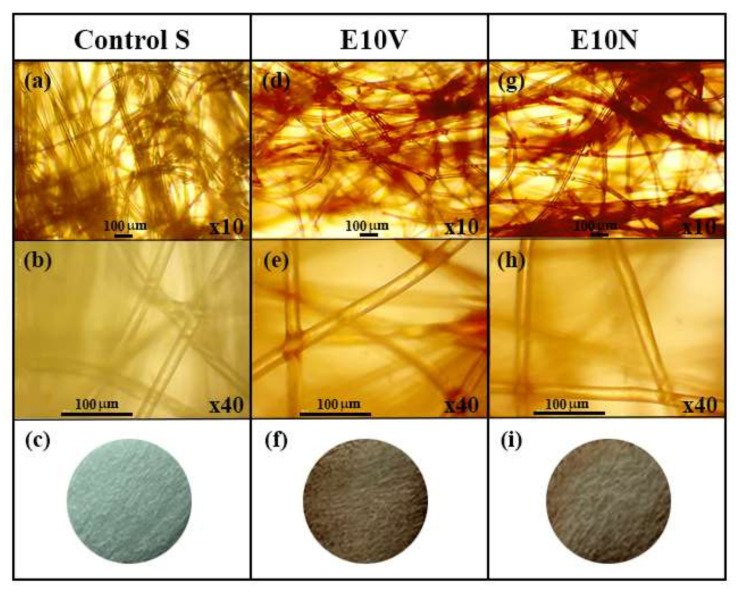
Optical microscopy images at two magnifications (×10 and ×40) and the macroscopic photographs of the neat non-woven fabric (Control S) before (**a**–**c**) and after the treatment with the VITAFAIR cranberry extract (E10V) (**d**–**f**) or the NUTRIBIOLITE cranberry extract (E10N) (**g**–**i**).

The optical microscopy images at two magnifications and the macroscopic photographs of the non-woven fabrics treated with and without cranberry extracts are shown in Figure 1.

**Figure 2:**
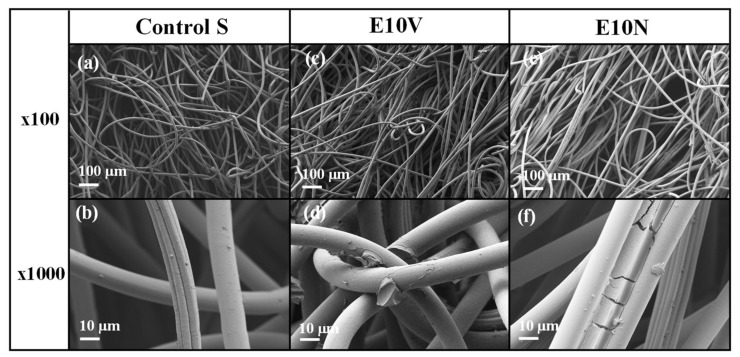
High-resolution field-emission scanning electron microscope (HR-FESEM) images of the non-woven fabric before (**a**,**b**) and after the treatment with the VITAFAIR cranberry extract (E10V) (**c**,**d**) or the NUTRIBIOLITE cranberry extract (E10N) (**e**,**f**).

Both optical microscopy images and macroscopic photographs (Figure 1c,f,i) show that only a very thin reddish coating of cranberry extract is formed onto the fibers of the non-woven fabrics. In addition, the surface morphology of the fabric samples was analyzed by electron microscopy (Figure 2). Before treatment, the fabrics present a smooth surface, with some little particles on the surface which may have arisen due to the spinning process (Figure 2a,b). After the treatment with the extracts, the fibers’ morphology and the overall porosity of the non-woven fabrics do not change substantially (Figure 2c,e), in good agreement with the results obtained from optical microscopy. The ×1000 magnification images show the fibers coated with the extracts (Figure 2d,f). The coating can be clearly observed (see Figure 2f), forming a layer that wraps the fibers. The surface of the fibers remains smooth, although a greater quantity of small particles deposited on the surface can be observed, probably due to the formation of small aggregates during the drying process after the dip-coating with the two cranberry extracts. Therefore, no substantial change of porosity or fiber arrangement is observed, which suggests no change of breathability or bacterial filtration efficiency required for certain infection prevention clothing applications.

### 2.2. Physico-Chemical Characterization

**Figure 3:**
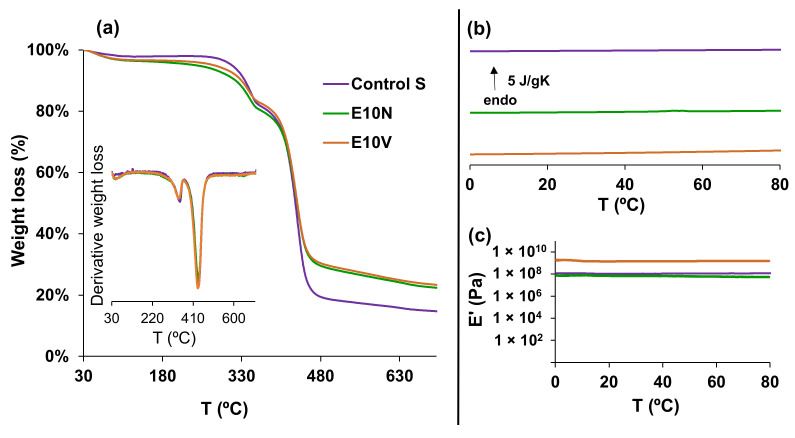
(**a**) Thermogravimetry (TGA) analysis. Weight loss and temperature derivative of the weight loss versus temperature. (**b**) Differential scanning calorimetry (DSC). Normalized heat flow on heating at 20 °C/min. (**c**) Dynamic mechanical thermal analysis (DMTA). Storage modulus (E′) vs. temperature at 1 Hz.

The thermal degradation profile of the neat non-woven sample (Control S) and the samples after dip-coating with the cranberry extracts (E10N and E10V), analyzed by thermogravimetry can be observed in Figure 3a. After an initial loss weight at temperatures below 100 °C, probably due to the evaporation of residual moisture, the degradation process began at temperatures above 220 °C and occurred in three stages. The degradation profile in the first and the second stage, between 220–360 °C and 360–450 °C, respectively, did not show large differences between the fabrics treated with the extracts and the untreated fabrics. However, at temperatures above 450 °C, the weight loss was approximately 10% lower in the samples treated with the cranberry extracts. The residual weight at the end of the assay (700 °C) remained with the same difference (10%) between the treated fabrics and the untreated fabric. In addition, the calorimetry results on heating, depicted in Figure 3b, showed that the treatment with the cranberry extracts did not affect the thermal behavior in a range of temperatures (from 0 to 80 °C) suitable for their application in infection prevention clothing.

Dynamic mechanical thermal analysis (DMTA) was also carried out between 0 and 80 °C to study the mechanical properties. These results show that the treatment with NUTRIBIOLITE extract (E10N) hardly modified the storage modulus of the untreated fabric. However, the storage modulus increased from 1.1 × 10^8^ ± 2.8 × 10^6^ Pa to 1.52 × 10^9^ ± 1.37 × 10^8^ Pa after the treatment with the VITAFAIR extract (E10V). These different effects of the treatments with the NUTRIBIOLITE and VITARFAIR extracts on the mechanical properties could be related to the different additives present in both commercial products.

These results provide evidence that the treatment with the cranberry extracts affects both the thermal and mechanical performance of the fabric, although not in the same manner.

### 2.3. Antiviral Results

**Figure 4:**
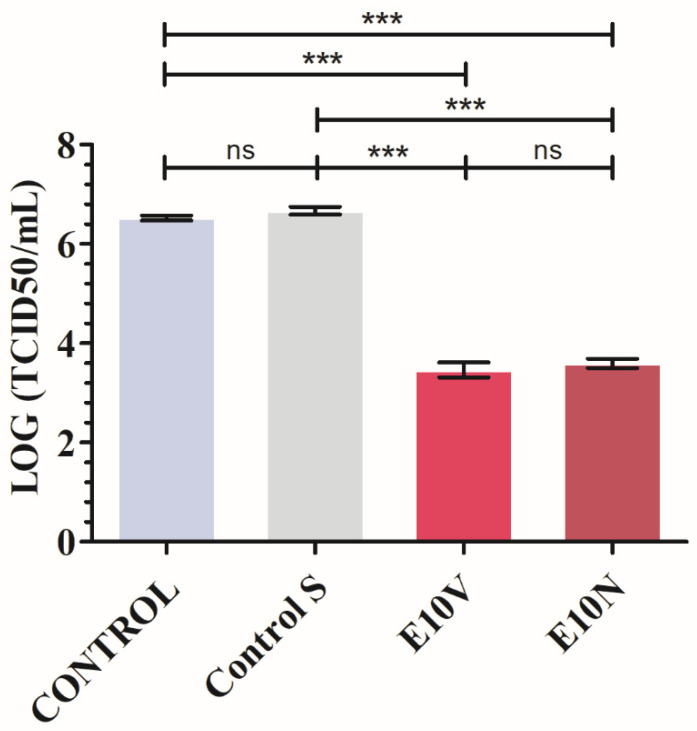
Reduction of infectious titers of SARS-CoV-2 after 1 min of contact evaluated by the median tissue culture infectious dose per mL (TCID50/mL) method. Viruses without being in contact with any fabric (CONTROL), untreated non-woven fabric (Control S), and non-woven fabric with a biofunctional coating of cranberry extracts of VITAFAIR (E10V) or NUTRIBIOLITE (E10N) at 1 min of viral contact. Three independent replicates (*n* = 3) were performed. Significant differences with respect to control were determined by one-way ANOVA with Tukey’s correction for multiple comparisons: *** *p* > 0.001; ns, not significant. Data are represented as means ± SD.

The results achieved with SARS-CoV-2 after 1 min of contact with the non-woven fabric treated with cranberry extracts and the controls are shown in Figure 4.

**Figure 5:**
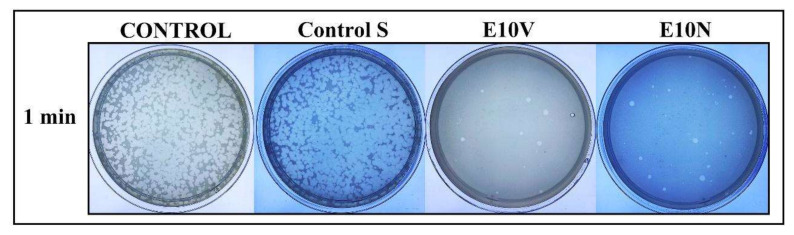
Bacteriophage phi 6 viability determined by the double-layer method. Titration images of undiluted samples for control, untreated non-woven fabric (Control S), and cranberry extract samples with biofunctional coating of cranberry extract of VITAFAIR (E10V) or NUTRIBIOLITE (E10N) at 1 min of viral contact. These images show that after the virus is in contact with the fabrics E10V and E10V for 1 min, most of them are inactivated, reducing very significantly the infection capacity (white spots).

By using non-woven fabric E10V and E10N, the median tissue culture infectious dose (TCID50) value was significantly reduced by a percentage of 99.91 ± 0.02 and 99.88 ± 0.02, respectively. Prolonged contact of SARS-CoV-2 with non-woven fabric E10V and E10N may allow more virus inactivation. Similar experiments would be needed using other SARS-CoV-2 variants such as Delta, Kappa and Lambda. Therefore, the non-woven fabric E10V and E10N showed strong antiviral activity against SARS-CoV-2. The antiviral results performed with the bacteriophage phi 6 are shown in Figure 5.

**Figure 6:**
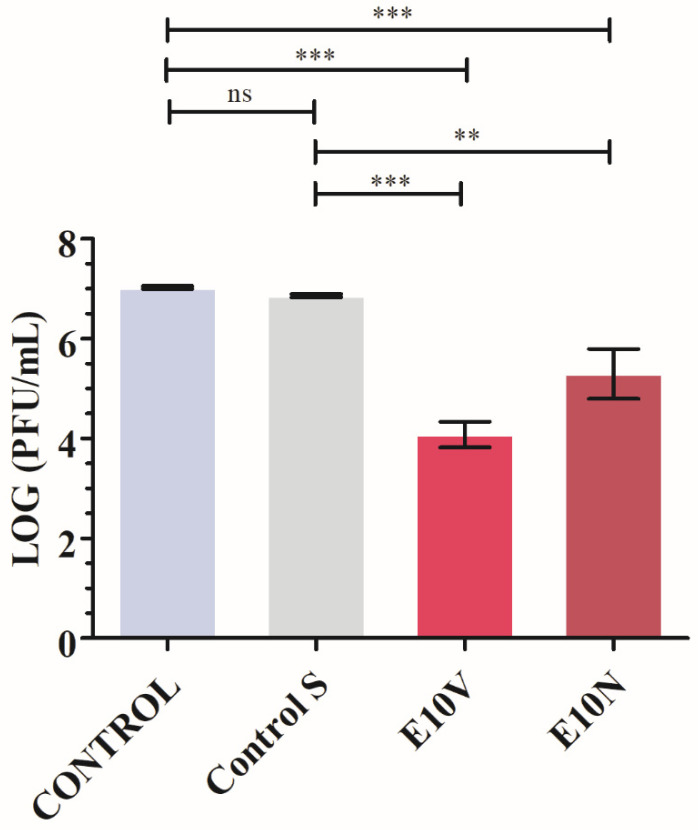
Reduction of infection titers of the bacteriophage phi 6 in logarithm of plaque-forming units per mL (log(PFU/mL)) and in PFU/mL measured by the double-layer method. Bacteriophages without being in contact with any fabric (CONTROL), untreated non-woven fabric (Control S), and non-woven fabric with biofunctional coating of cranberry extract of VITAFAIR (E10V) or NUTRIBIOLITE (E10N) at 1 min of viral contact. Three independent antiviral tests were performed in two different days (*n* = 6). Significant differences with respect to control were determined by one-way ANOVA with Tukey’s correction for multiple comparisons: *** *p* > 0.001; ** *p* > 0.01; ns, not significant.

Thus, the bacteriophage phi 6 loses infectivity after being in contact with both types of antimicrobial fabrics for 1 min and thus very few plaques are observed on the bacterial lawns. However, a similar amount of plaques is observed in the control and untreated fabric on the bacterial lawns after the same time of contact (see Figure 5). The phage titers in PFU/mL of each type of sample were calculated and compared with the control in log reductions (see Figure 6).

Figure 6 shows that the titers obtained by contacting the phages with the Control S for 1 min are similar to the CONTROL. However, the phages in contact with the fabrics containing cranberry extract (E10V and E10N) for 1 min displayed a statistically significant phage infectivity reduction of 99.87 ± 0.07% and 97.39 ± 1.95%, respectively. Therefore, these results of SARS-CoV-2 and the bacteriophage phi 6 clearly show the potent antiviral activity of the cranberry extracts independently of the commercial brand confirming our first hypothesis of antiviral activity.

Cranberry extracts are well known for their pharmacological potential [42,43,44,45]. Furthermore, the natural product proanthocyanidins present in the cranberry extracts are widely used as cosmetic, suggesting a potential use as disinfectant for external use [46]. Su et al. showed the antiviral activity of the cranberry juice and PACs against different virus, including human enteric virus surrogates, murine norovirus, feline calicivirus, bacteriophage MS2 (ssRNA), and bacteriophage phiX-174 (ssDNA) [47]. They demonstrated a very significant reduction of virus titers after 1 h of viral contact with the cranberry juice and the PACs. However, in the present study, we have shown the inactivation of SARS-CoV-2 and bacteriophage phi 6 (about 99% of viral inactivation) in much lower time (1 min), because the goal of this project was to achieve a bio-based coating capable of inactivating enveloped viruses such as SAR-CoV-2 and bacteriophage phi 6 as fast as possible to provide higher protection in their application as infection prevention clothing such as face masks. This fast antiviral activity can be attributed to the high surface/volume ratio of these composite materials formed by coated fibers.

Regarding the antiviral mechanism of action, Mirandola et al. attributed the antiviral activity of the cranberry extracts against Hazara virus to the target of early stages of the viral replication cycle, including the initial adsorption to target cells [48]. There are different hypotheses about how cranberry extracts or the PACs affect viruses. The PACs could act by binding and destroying the viral capsid structure or could cause inhibition of key enzymes involved in viral replication [49]. A recent study on the discovery of SARS-CoV-2 channel inhibitors as antiviral candidates, has shown that PACs directly bound to 2-E channel with binding affinity (KD) of 22.14 μM in surface plasmon resonance assay [46]. In addition, some studies have shown that PACs exposure prevents some bacteriophages from attaching to their bacterial host [50].

### 2.4. Antibacterial Results

**Figure 7:**
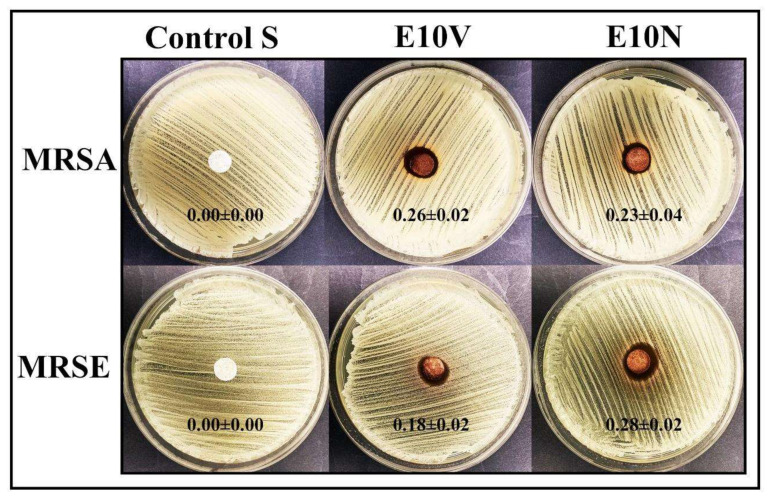
Agar disc diffusion tests with the methicillin-resistant *Staphylococcus aureus* (MRSA) and the methicillin-resistant *Staphylococcus epidermidis* (MRSE). Control sample (Control S), and the fabrics treated by dip-coating with the two cranberry extracts: VITAFAIR (E10V) and NUTRIBIOLITE (E10N). Aerobic incubation for 24 h at 37 °C. The normalized widths of the antibacterial halos (*nw_halo_*) calculated with equation (1) are shown as mean ± standard on each image. These images show how the E10V and E10V fabric disks produce a significant bacterial inhibition zone around them after 24 h of bacterial culture.

The antibacterial results obtained by the disc diffusion test are shown in Figure 7.

Cranberry extracts and PACs can help to prevent and reduce the recurrence of urinary tract infections by decreasing bacterial adhesion [51]. Figure 7 shows that the E10V and E10N fabric possess potent antibacterial activity against both MRSA and MRSE pathogens. Treatment of *S. aureus* with cranberry extracts revealed a transcriptional signature typical of PG-acting antibiotics, that reveals inhibition of bacterial peptidoglycan biosynthesis [52]. Furthermore, it is well-known that cranberry extracts and PACs affect more Gram-negative bacteria than Gram-positive bacteria because they can more easily damage their membranes and walls [51].

Therefore, both non-woven fabrics with biofunctional coatings of cranberry extract showed potent antibacterial properties against MRSA and MRSE confirming our second hypothesis of antibacterial activity.

### 2.5. In Vivo Toxicity

**Figure 8:**
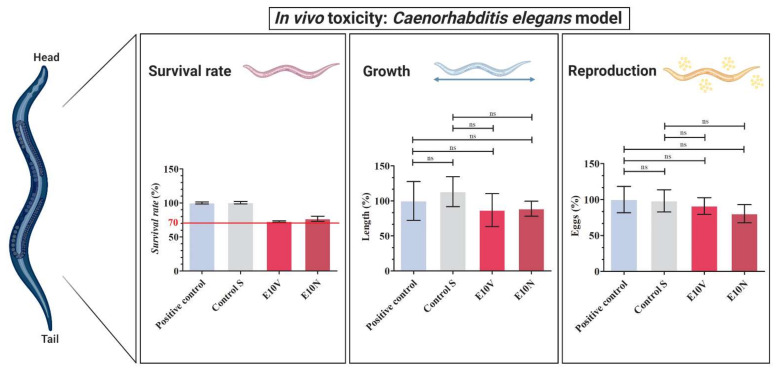
In vivo toxicity in *Caenorhabditis elegans* model: survival rate, growth and reproduction after exposure with the extracts of the untreated non-woven fabric (Control S), the extracts of the non-woven fabric treated with the VITAFAIR cranberry extract (E10V) and the extracts of the non-woven fabric treated with the NUTRIBIOLITE cranberry extract (E10N) with respect to the positive control (100%). Five independent replicates (*n* = 5) were conducted for this assay. Results are shown as mean ± standard; Significant differences with respect to control were determined by one-way ANOVA with Tukey’s correction for multiple comparisons: ns, not significant.

In vivo toxicity was studied with the *C. elegans* model, which is a nematode that can be handled at low cost using standard in vitro techniques [53]. Two thirds of human proteins have *C. elegans* homologs, and about 80% of genes for human inborn errors of metabolism have *C. elegans* homologs [54,55]. It has also been described that *C. elegans* alimentary system has many facets that are comparable to that of mammals [56,57] and over 70% of human lipid genes are conserved in *C. elegans*, and 20% of *C. elegans* lipid genes are orthologs of human metabolic disease genes [58]. Moreover, *C. elegans*’ genomics has been used to study human development and disease [54]. Furthermore, it presents fewer ethical problems and shares many genes and signaling pathways with humans. Unlike cytotoxicity assays, *C. elegans* toxicity tests provide data from a whole animal with intact and metabolically active digestive, reproductive, endocrine, sensory and neuromuscular systems [59]. Thus, survival rate, growth and reproduction of *C. elegans* was analyzed after an exposure of 24 h to the extracts of the antimicrobial fabrics (see Figure 8). An exposure of 24 hours is long enough for their application for face masks manufacture because they should be worn for no longer than 4 h according to the recommendations of the World Health Organization (WHO) [60].

**Figure 9:**
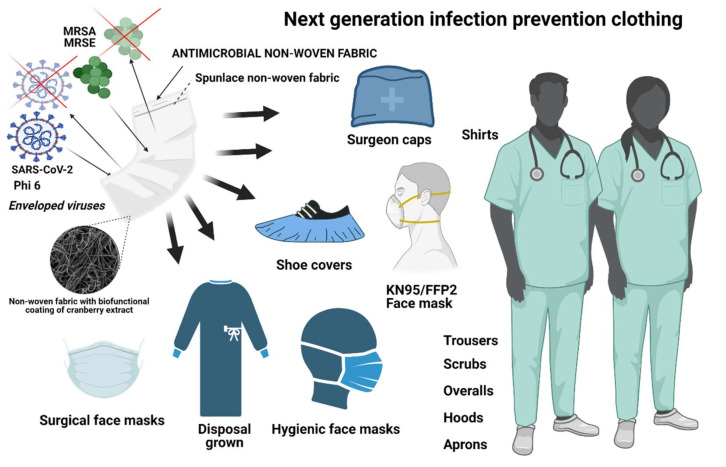
Antiviral infection prevention clothing produced with the developed antiviral low-cost technology capable of inactivating enveloped viruses such as SARS-CoV-2 and phi 6, and the methicillin-resistant bacteria *Staphylococcus aureus* (MRSA) and methicillin-resistant *Staphylococcus epidermidis* (MRSE) bacteria. Created with BioRender.com.

Both antimicrobial fabrics (E10V and E10N) showed a survival reduction lower than 30% in comparison with *C. elegans* in the optimal growth conditions (positive control). Furthermore, the extracts of the E10V and E10V non-woven fabrics did not show any effect on growth or reproduction showing the nematodes similar length and number of eggs after 24 h of exposure. Therefore, these composite materials can be considered non-toxic, especially for infection prevention clothing applications where the materials are used outside the body. It is of note that the antimicrobial non-woven fabrics developed in this study have been produced with cranberries, which are natural and biodegradable products that can be easily grown and thus provide great promise in the fight against SARS-CoV-2. Thus, the fabrication procedure presented here can be used to produce many types of biobased face masks and other next-generation infection prevention clothing such as caps, scrubs, shirts, trousers, disposable gowns, overalls, hoods, aprons and shoe covers to inactivate enveloped viruses such as SARS-CoV-2 (see Figure 9).

## 3. Materials and Methods

### 3.1. Fabric Preparation

Cranberry extracts with a concentration of 10% *w*/*v* were prepared using ethanol as extracting solvent and two different types of commercial cranberry powders of *Vaccinium macrocarpon*: VITAFAIR (Whitewall GmbH, Berlin, Germany) and NUTRIBIOLITE (Uritractin, Valladolid, Spain). According to the VITAFAIR and NUTRIBIOLITE manufacturers, the cranberry powders have a proanthocyanidins content of 25 and 30% *w*/*w*, respectively. Thus, 10 g of cranberry powder was mixed with 100 mL of absolute ethanol (≥99.8%, AnalaR Normapur, VWR chemicals, Radnor, PA, USA) under magnetic stirring for 30 min at 24 ± 1 °C sealed with parafilm. After that, the extract was left overnight to decant the solid phase. After 12 h, the supernatant was centrifuged at 10,000 r.p.m (16,993× *g*) in a Centrifuge Heraeus Megafuge 16R (Thermo Scientific, Waltham, MA, USA) for 30 min to ensure complete phase separation of the liquid cranberry extract from the solid phase. After centrifugation, extract was left overnight to decant possible solid phase remaining. The day after, supernatant was removed with a 25 mL serological pipette (LABCLINICS, Barcelona, Spain) and filtered with a non-sterile 0.2 μm filter. After filtering, extract was centrifuged again at 10,000 r.p.m. (16,993× *g*) for 30 min to ensure complete solid phase separation from the liquid phase. Thus, commercial non-woven spunlace fabric (NV EVOLUTIA, Valencia, Spain) was prepared in the form of discs of approximately 10 mm in diameter. After that, they were treated with the two different cranberry extracts by the dip-coating method [61]. Thus, the non-woven fabrics were immersed in the cranberry extracts (10% *w*/*v*) solutions for 30 min at 24 ± 1°C sealed with parafilm. After that, the prepared samples were dried at 60 °C for 48 h to solidify the physically absorbed cranberry extract to form the coating and ensure complete evaporation of the ethanol phase. The non-woven fabrics treated with VITAFAIR and NUTRIBIOLITE cranberry extracts will be named hereafter as E10V and E10N, respectively. The discs were sterilized by ultraviolet radiation for one hour per side. Discs prepared from the non-woven fabrics treated with only the absolute ethanol (≥99.8%, AnalaR Normapur, VWR chemicals, Radnor, PA, USA) solvent under magnetic stirring for 30 min at 24 ± 1 °C (Control S) were prepared as reference material.

### 3.2. Physicochemical Characterization

#### 3.2.1. Fabric Morphology

The morphology of the non-woven fabrics treated with and without cranberry extracts was observed by optical microscopy (Motic BA410E) and was photographed at 10× and 40× magnifications with the Moticam 580 5.0MP. The images were processed by the Motic Images Plus 3.0 software (Motic, Barcelona, Spain). Macroscopic photographs of the fabrics were also performed with a 64MP Xiaomi camera with a Sony IMX682 sensor and a f/1.89 opening. Scanning electron microscopy was used to analyze in greater detail the morphology of the fibers of the non-woven fabric (porous structure). Untreated and treated non-woven fabrics samples were observed at a magnification of ×100 and ×1000 with a GeminiSEM 500 high-resolution field-emission scanning electron microscope (HR-FESEM) (Zeiss-Oxford Instruments, Abingdon, UK) with an accelerating voltage of 1.5 kV. The samples were previously coated with a platinum layer by an EM MED020 sputter coater (Leica).

#### 3.2.2. Thermal Degradation and Thermal Behavior

Thermal gravimetric analysis (TGA) was carried out on a Mettler Toledo TGA 2 (SF system) (Metter Toledo, Columbus, USA). Dried samples (between 5–10 mg) were placed on the balance and the weight loss of the sample was measured as a function of temperature. The samples (~10 mg) were heated from 25 to 700 °C at a heating rate of 20 °C/min.

Differential scanning calorimetry (DSC) was performed on a PerkinElmer DSC 8000 (PerkinElmer, Waltham, USA) under a flowing nitrogen atmosphere on samples between 5 and 10 mg. Vacuum-dried samples were subjected to a cooling scan down to 0 °C, followed by a heating scan up to 80 °C, both at a rate of 20 °C/min.

#### 3.2.3. Mechanical Properties

Dynamic mechanical thermal analysis was carried out with a DMA 8000 (PerkinElmer, Waltham, USA) at a frequency of 1 Hz on sample bars (20 × 5 × 0.01 mm). The temperature dependence of the storage modulus (E′), loss modulus (E″), and loss tangent (tan δ) was measured in the temperature range 0 to 80 °C at a heating rate of 3 °C/min under nitrogen atmosphere. Three independent experiments were performed to ensure reproducible results.

### 3.3. Antiviral Test against SARS-CoV-2

The SARS-CoV-2 (SARS-CoV-2/Hu/DP/Kng/19-027) was provided by Dr. Tomohiko Takasaki and Dr. Jun-Ichi Sakuragi (Kanagawa Prefectural Institute of Public Health). A volume of 100 μL of a SARS-CoV-2 suspension in phosphate buffered saline (PBS) was added to each disc at a titer dose of 5.0 × 10^6^ TCID50/disc, and then incubated for 1 min at room temperature. A volume of 900 μL PBS was added to each disc (Control S, E10V and E10N), and then vortexed for 5 min. After that, each disc was vortexed for 5 min. Briefly, TMPRSS2/Vero cells (JCRB1818, JCRB Cell Bank, Osaka, Japan) were cultured with the Minimum Essential Media (MEM, Sigma-Aldrich, St. Louis, MO, USA) containing 5% fetal bovine serum (FBS), 1% penicillin/streptomycin (P/S) on the 96-well plates (Thermo Fisher Scientific, Waltham, MA, USA). Samples were serially diluted 10-fold from 10^−1^ to 10^−8^ in the MEM containing 5% FBS and 1% P/S. Dilutions were placed onto the TMPRSS2/Vero cells in triplicate and incubated at 37 °C for 96 h. Cytopathic effects were evaluated under a microscope. TCID50/mL were calculated using the Reed-Muench method. The SARS-CoV-2 infection experiments were conducted at a Biosafety Level 3 laboratory at Kyoto University. Three independent replicates (*n* = 3) were performed for this assay.

### 3.4. Antiviral Test against Bacteriophage Phi6

*Pseudomonas syringae* (DSM 21482) is the host of the bacteriophage phi 6. This Gram-negative bacterium was purchased from the Leibniz Institute DSMZ–German Collection of Microorganisms and Cell cultures GmbH (Braunschweig, Germany). This microorganism was cultured in solid tryptic soy agar (TSA, Liofilchem, Teramo, Italy) and, after that, in liquid tryptic soy broth (TSB, Liofilchem, Teramo, Italy) at a speed of 120 r.p.m. at 25 °C. The Leibniz Institute DSMZ–German Collection of Microorganisms and Cell Cultures GmbH specifications were followed to propagate the bacteriophage phi 6 (DSM 21518). The antiviral assay was performed with a dispersion of 50 μL of TSB with phages placed onto each sample (Control S, E10V and E10N) at a titer of approximately 1 × 10^6^ plaque-forming units per mL (PFU/mL) and incubated for 1 min. A falcon tube was used to place each disc with 10 mL TSB to be sonicated and vortexed for 5 and 1 min, respectively, at ambient temperature (24 ±1 °C). Phage titration by serial dilutions of each falcon sample was performed and 100 μL of each phage dilution was mixed with 100 μL of the bacterial host at OD_600nm_ = 0.5. The infective capacity of the bacteriophage phi 6 was analyzed based on the double-layer method [62]. Thus, a volume of 4 mL of top agar (TSB + 0.75% bacteriological agar, Scharlau) and 5 mM calcium chloride (Sigma-Aldrich, St. Louis, MO, USA) were added to the phage dispersion mixed with the bacteria, and then poured on TSA plates for incubation for 18–24 h in a refrigerated oven at 25 °C. Phage titers in PFU/mL of each sample were compared with control (CONTROL), which consisted of 50 μL of phage added directly to the bacterial culture without being in contact with any type of disc and without the sonication/vortexing treatment. The antiviral activity of the discs coated with cranberry extract or not was determined at 1 min of contact with the bacteriophage phi 6 in log reductions of titers. It was made sure that the sonication/vortexing treatment did not affect the infectious activity of the bacteriophage phi 6 and that the residual disinfectants of the titrated samples did not interfere with the titration process. Three independent antiviral tests were performed in two different days (*n* = 6) to ensure reproducibility.

### 3.5. Antibacterial Tests

Lawns of MRSA, COL [63], and MRSE, RP62A [64], were used to perform the antibacterial assays by the agar disc diffusion tests [65,66] at a concentration of approximately 1.5 × 10^8^ colony forming units per mL (CFU/mL) in tryptic soy broth, and then cultivated on trypticase soy agar plates. Incubation was performed aerobically at 37 °C for 24 h with the sterilized samples treated with (E10V and E10N) and without cranberry extract (Control S) placed upon them. The inhibition zone (or halo) was normalized according to Equation (1) [65].

(1) $$nw_{halo}=\frac{\frac{d_{iz}-d}{2}}{d}$$

In this equation, *nw_halo_* expresses the normalized width value of the antibacterial inhibition zone, *d_iz_* indicates the diameter of the inhibition zone and the term *d* represents the diameter of the disc. The diameter of the disc was measured by image software analysis (Image J, Wayne Rasband (NIH), USA, Bethesda, MD, USA). Three independent antibacterial assays were performed in two different days (*n* = 6) to provide reproducible results.

### 3.6. In Vivo Toxicity Tests

In vivo toxicity was studied in the *Caenorhabditis elegans* model. Extractions from the non-woven fabrics with (Control S) and without (E10V and E10N) cranberry extract treatment were accomplished following the ISO-10993 standard recommendations. Thus, spunlace fabrics treated with and without cranberry extracts were subjected to sterilization under ultraviolet light (1 h per side). A 6-well plate was used to place every piece of fabric into a well with 2 mL of potassium medium (2.36 g potassium chloride, 3 g sodium chloride in 1 L distilled water, autoclaved). A volume ratio of 0.1g/mL was selected according to the ISO-10993 that recommends this rate for irregular porous materials of low density such as textiles. After incubating for 24 h at 25 °C, extracts were collected in 1.5 mL eppendorf tubes. The worms were maintained and propagated on OP50 *E. coli* seeded nematode growth medium (NGM) prepared according to Stiernagle, T. [67] at 25 °C. An N2 strain was used in these experiments, provided by the *Caenorhabditis* Genetics Center (CGC, Minneapolis, MN, USA). The worms and eggs were washed off NGM plates using 5 mL of distilled water and collected in 15 mL falcon tubes to prepare an L1 stage-synchronized *C. elegans* population. Tubes were centrifuged at 1300 r.p.m. (2209× *g*) for 3 min and supernatant was moved away. *C. elegans*’ pellet was resuspended in 100 μL of dH_2_O and transferred to eppendorf tubes adding 700 μL of a 5% bleaching solution. This mixture was incubated for 15 min while vortexing every 2 min. After the last vortexing procedure, eppendorf tubes were centrifuged at 700× *g* for 3 min. Supernatant was moved away, and the pellet was washed in 800 μL of dH_2_O. This step was carried out two more times. After the last washing step, the pellet was resuspended in 100 μL of dH_2_O and transferred to NGM plates seeded with 100 μL of an OP50 *E. coli* culture. Eggs were incubated for 72 h at 25 °C. Centrifugation at 1300 r.p.m. (2209× *g*) for 3 min was performed to pellet the L1 staged populations and subsequent resuspension in 3 mL of potassium medium was performed. A 48-well plate was used to prepared wells with 62.5 μL of a 1:250 suspension of cholesterol (5 mg/mL in ethyl alcohol) in sterile potassium medium, 62.5 μL of a 50× concentrated OP50 *E. coli* culture with an OD of 0.9, pelleted by centrifugation at 4000 r.p.m. (6797× *g*) for 10 min and resuspended in potassium medium, 115 μL of potassium medium and 250 μL of the pertinent cranberries extract. A volume of K medium containing 50–100 worms was then added. 48-well plates were sealed with parafilm and placed in an orbital shaker at 25 °C and 120 r.p.m. for 24 h. In order to determine the survival rate of *C. elegans*, the volume of each well of the 48-well plates was divided in 10 drops of 50 μL and placed under the microscope (Motic BA410E including Moticam 580 5.0MP) in order to count the number of living *C. elegans* and deceased ones. The growth in the medium and in bleach were used as a positive and negative control, respectively. To analyze reproduction, three worms were placed into a new OP50 seeded NGM plate and allowed to lay eggs for 48 h. Then, eggs were counted by using the microscope. Growth was assessed in heat-killed samples by measurement the body length in a picture taken under the microscope with Motic Images Plus 3.0 software. Five independent replicates (*n* = 5) were conducted for this assay.

### 3.7. Statistical Analysis

The ANOVA statistical analysis followed by Tukey’s post hoc test (* *p* > 0.05, *** *p* > 0.001) was performed using the GraphPad Prism 6 software (GraphPad Software Inc., San Diego, CA, USA). Three independent replicates (*n* = 3), three independent antiviral tests in two different days (*n* = 6) and five independent replicates (*n* = 5) were performed for the TCID50 assay, the double-layer method, and the in vivo assay, respectively.

## 4. Conclusions

Two different non-woven fabrics have been developed with two types of commercial biobased cranberry extracts by dip-coating. Both composite fabrics showed no toxicity in *a Caenorhabditis elegans* in vivo model and high antiviral activity (about 99% of viral inactivation) against both enveloped viruses SARS-CoV-2 and bacteriophage phi 6 after just one min of contact. Since cranberry extracts have also exhibited antiviral activity against other enveloped viruses such as HSV-1, HSV-2, and IFV, the idea that PACs produce strong alterations of their envelope glycoproteins achieving their inactivation is becoming more and more consistent. Furthermore, the non-woven antiviral fabrics showed potent antimicrobial activity against the Gram-positive methicillin-resistant bacteria *Staphylococcus aureus* and *Staphylococcus epidermidis*. Optical and electron microscopy did not show any significant changes of morphology of the neat fabric after the dip-coating treatment. However, the treatment with the extracts somehow affected both the thermal and mechanical performance of the neat fabric depending on the type of commercial extract used. Therefore, further research could elucidate natural and biodegradable broad-spectrum treatments for the next generation of infection prevention clothing in the current COVID-19 pandemic and for future microbial threats.

## Data Availability

Data is contained within the article.
